# The Antibacterial Effectiveness of Propolis on Medical Screws

**DOI:** 10.7759/cureus.16278

**Published:** 2021-07-09

**Authors:** Lokman Kehribar, Hüseyin Sina Coşkun, Serkan Surucu, Mahmud Aydın, Mahir Mahiroğulları

**Affiliations:** 1 Orthopedics and Traumatology, Samsun Gazi State Hospital, Istanbul, TUR; 2 Orthopaedics and Traumatology, Samsun Ondokuz Mayıs University Faculty of Medicine, Samsun, TUR; 3 Orthopaedics and Traumatology, Horasan State Hospital, Erzurum, TUR; 4 Orthopedics and Traumatology, Haseki Education Research Hospital, Istanbul, TUR; 5 Orthopedics and Traumatology, Memorial Şişli Hospital, Istanbul, TUR

**Keywords:** propolis-coated screw, surface modification, biocompatible charged gel, biofilm layer, antimicrobial activities

## Abstract

Background

Medical screws are widely used in orthopedic surgery for fracture fixation. The antibacterial effectiveness of propolis is well known. In this study, we aimed to demonstrate the antibacterial effectiveness of medical screws coated with propolis.

Methodology

Between March 2019 and April 2020, we formed five experimental groups and investigated the antibacterial activities of different amounts of propolis and polymer-coated screws. *Staphylococcus aureus* was used to determine the antibacterial activity. Carbopol, chosen as the model polymer, was used to improve the adhesion of propolis to the screws. Agar diffusion test of surface-coated screws was used to evaluate the antibacterial effect.

Results

The mean zone diameters were 24.3 ± 1.1, 23.0 ± 0.8, 21.8 ± 1.6, 19.3 ± 0.6, and 20.2 ± 0.8 mm for IS-7.5, IS-5.0, IS-2.5, IS-P, and IS-P-7.5, respectively. The IS-7.5 group had the most antibacterial activities. The antibacterial activities of the medical screws determined using the agar diffusion method were significantly increased by the propolis coating on the screws. Our results showed that the propolis-coated screws had antibacterial activity against *S. aureus*.

Conclusions

As a result, we believe that the combination of gel and propolis is an effective method in increasing the antibacterial resistance of medical screws and preventing the formation of a biofilm layer of microorganisms.

## Introduction

The behavior of implants in biological environments is extremely important in terms of patient quality of life and treatment outcome [1]. The most important criteria to be considered during the design phase of implants used in orthopedics are biocompatibility, the ability to resist erosion by body fluids over time, and providing permanent osteointegration [2,3]. To improve the osteointegration feature of implants, surface modification to obtain a wettable and charged surface is a basic approach during implant development [4]. In the literature, surface modification methods have been classified as mechanical, chemical, and biochemical [5-7]. However, in recent years, anti-infective surface modifications of orthopedic implants have become the most needed implant coating technology to resist implant-related infections [8,9]. The traditional approach to preventing bacterial colonization on the implant is the implantation of antiadhesion or antibacterial agents. Some of these agents are antimicrobial peptides, silver nanoparticles, and antibiotic coatings [10,11].

Owing to its antibacterial properties and importance in nutrition, propolis has been a subject of studies in the literature [12,13]. It generally includes propolis wax [14], phenolic mixtures [15], and other minor ingredients such as sugar, amino acids, and powder. Various properties of propolis have been reported to show a beneficial role in not only biological environments but also material development strategies [16]. In addition, the anti-inflammatory, antioxidant, antibacterial, antiviral, and antifungal properties of propolis have been reported [17,18].

In this study, we aimed to evaluate the changes in the antibacterial resistance of medical screws in the presence of propolis, which is a promising biological material.

## Materials and methods

**Figure 1:**
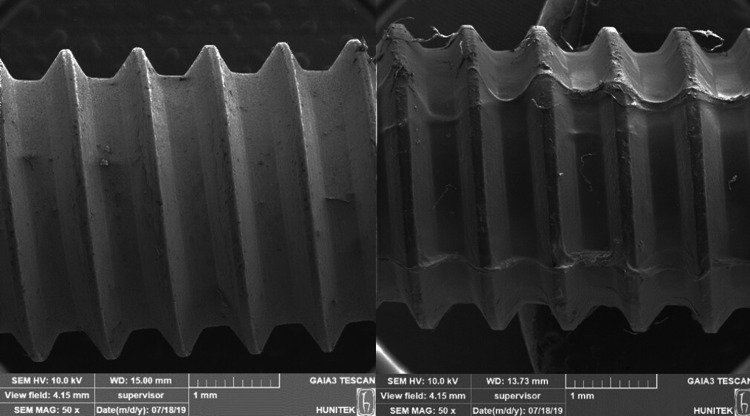
Scanning electron microscopic images of (A) noncoated and (B) coated medical screws (IS-7.5).

**Figure 2:**
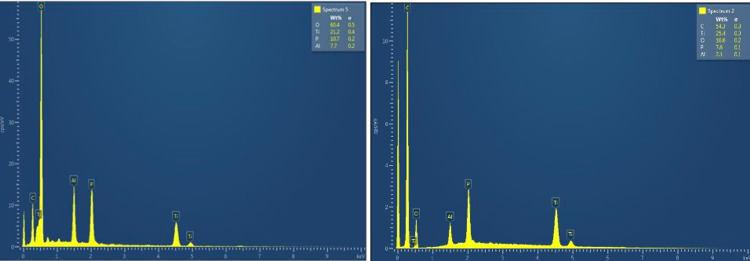
Electron dispersive spectroscopy atomic mappings of (A) noncoated and (B) coated medical screws (IS-7.5). C: carbon; Ti: titanium; O: oxygen; P: phosphorous; Al: aluminum

Between March 2019 and April 2020, we designed a series of experiments to show that medical screws gain antibacterial properties from propolis. Scanning electron microscopic images of uncoated and coated medical screws are shown in Figure 1. In the figure, in addition to the surface topography, the propolis coating layer on the medical screws is seen, which has changed the smoothness significantly. To confirm the surface coating process, we applied energy dispersive spectroscopy while taking element maps on the surface of the material (Figure 2). As can be seen in the figure, the number of carbon atoms originating from the polymer and propolis has increased greatly. Antibacterial examination of surface-modified screw implants was performed after the agar diffusion test (Kirby-Bauer). The antibacterial activity in each experimental group was compared with the penicillin-loaded standard disk.

Nitric acid (90%-309079), ethanol (99%-24102), Mueller-Hinton agar (105437), and tryptic soy agar (22091) were purchased from Merck (Darmstadt, Germany). Phosphate-buffered saline (10010023) was purchased from Thermo Fisher Scientific (Bleiswijk, The Netherlands). Antibiotic disks for comparison were purchased from Hardy Diagnostics (Santa Maria, CA, USA). *Staphylococcus aureus* (25923) was purchased from American Type Culture Collection (Manassas, VA, USA). Screw implants (Ti6Al4V ELI) were purchased from Sandvik Coromant (Sandviken, Sweden). Biocompatible charged carrier polymer (Carbopol 940) was purchased from Lubrizol (Brussels, Belgium). Propolis was a gift from a local honey producer, Hızır Öztürk (Murgul, Artvin, Turkey), and was harvested in September 2019.

Surface coating of implant screws

The loading of propolis on medical screws was achieved using carbopol as a carrier-charged polymer. For this purpose, 400 mg of the polymer was dissolved in 40 mL of ethanol solution (25% v/v) containing different amounts of propolis. Propolis amounts were adjusted to 2.5%, 5.0%, and 7.5% by mass of carbopol amount. The carbopol and propolis solutions were homogenized at 25°C for 30 minutes and stirred at 500 rpm for two days. To polish the screw surface, the implant parts of approximately the same dimensions were immersed in a concentrated HNO_3_ solution placed in a boiling water bath for five minutes [19,20]. Then, the implant parts were rinsed with deionized water three times to remove acidic residues and were separately placed in 24-well plates until completely dried. Meanwhile, 2 mL of the solutions containing carbopol and propolis were added into the cells of the well plates in sequential order of the following implant screws (IS): IS-7.5, IS-5.0, IS-2.5, IS-P, and IS-P-7.5 (Table 1). The screws were driven into the cells and allowed to interact with the solutions for 15 minutes to achieve surface coating. Finally, the screws were separated from the solutions and transferred into a clean well plate and dried in an oven at 25°C overnight. Two control experimental sets were designated as IS-P and IS-P-7.5, which included only polymer and propolis, respectively, to evaluate the effects of the polymer without propolis and propolis without the polymer.

**Table 1:** Description of the surface-coated screws. *The experiment was repeated three times for each group for statistical purposes.

Implant screw	Experimental group*
IS-7.5	Screws coated with a polymer (10 mg/mL in ethanol) and propolis 7.5% of the mass of the polymer
IS-5.0	Screws coated with a polymer (10 mg/mL in ethanol) and propolis 5.0% of the mass of the polymer
IS-2.5	Screws coated with a polymer (10 mg/mL in ethanol) and propolis 2.5% of the mass of the polymer
IS-P	Screws coated only with a polymer (10 mg/mL in ethanol)
IS-P-7.5	Screws coated only with propolis of the same amount as that in IS-7.5

Antimicrobial activity

To compare antimicrobial activities, the test disk (P 10) with penicillin (10 U) was used as the standard antimicrobial agent (Hardy Diagnostics, Santa Maria, CA, USA). The bacterial strain was incubated on nutrient agar plates at 37°C for 24  hours. Mueller-Hinton agar is a growth medium used to determine the susceptibility of microorganisms to antimicrobial agents. For this, 20.4 g of agar was dissolved in 600 mL of distilled water by heating till boiling to dissolve completely. Then, the medium was sterilized by autoclaving at 121°C for 15 minutes and cooled to 45-50°C. The medium (20 mL) was added to each petri dish. Meanwhile, the screws were rinsed in 5 mL of phosphate-buffered saline solution (pH 7.4, 10 mM) and incubated in a 48-well plate for 12 hours. The extracts (20 µL) from five different experimental groups were added to the blank test disks. The dried disks with the propolis extracts were placed on the culture maintaining proper distance. After placing the disks, the media were kept in an incubator at 37°C for 48 hours. The measurements were studied in three replicates, and zone diameter measurements were applied in triplicates.

## Results

The zone diameter measurements for the five experimental groups are summarized in Table 2. In each group, the zone diameter of the standard disk was 30.0 mm, which indicated high precision and accuracy between each petri dish. As summarized in the table, the propolis-loaded gels (IS-7.5/5.0/2.5) showed significant antibacterial activity compared to the control sets (IS-P/P-7.5). These results show that the enhanced antibacterial activity of the medical screws might have been caused by the propolis-loaded gels.

**Table 2:** Kirby-Bauer zone diameter measurements for medical screws The zone diameter of the penicillin disc was 30.0 mm for each sample. The zone diameter of the blank disc containing no penicillin and propolis extract was 16.9 mm.

Group	Zone diameter, mm	Standard deviation, ±SD
IS-7.5	24.3	1.1
IS-5.0	23.0	0.8
IS-2.5	21.8	1.6
IS-P	19.3	0.6
IS-P-7.5	20.2	0.8

The mean values of the zone diameters were 24.3 ± 1.1, 23.0 ± 0.8, 21.8 ± 1.6, 19.3 ± 0.6, and 20.2 ± 0.8 mm for IS-7.5, IS- 5.0, IS-2.5, IS-P, and IS-P-7.5, respectively. The standard deviation for each set ranged from 3.2% to 7.2%, which indicated that the antibacterial tests and their results were at an analytically acceptable level.

The median zone diameters for each set were 24.0, 22.5, 21.8, 19.5, and 20.1 mm for IS-7.5, IS- 5.0, IS-2.5, IS-P, and IS-P-7.5, respectively, supporting the above discussion, as they were close to and/or the same as the mean values. The variance between the results of each set was also calculated to evaluate the precision and accuracy of the experiments and were 1.1, 0.5, 2.1, 0.3, and 0.5 for IS-7.5, IS-5.0, IS-2.5, IS-P, and IS-P-7.5, respectively. These results indicate that the surface coating, propolis extraction/release, and Kirby-Bauer antibacterial resistivity test were performed with high precision and accuracy. Moreover, the addition of propolis into the gel coating mixture improved the antibacterial property of the medical screws, whereas the antibacterial effects of the gel without propolis (IS-P) and propolis without gel (IS-P-7.5) were limited.

## Discussion

*S. aureus* is a common pathogen in soft tissue and musculoskeletal infections [21]. In addition, staphylococcal infections are resistant to antibiotics. In our study, the antimicrobial activities of the propolis-coated medical screws were studied against a gram-positive bacteria (*S. aureus* ATCC 25923) chosen as a model bacterial species because it is the most problematic and frequently contaminating species in surgical sites.

Propolis and active compounds have shown in vitro antimicrobial activities against gram-positive and gram-negative species [22-24]. In our study, the results indicated that the combination of gel and propolis is necessary for achieving proper improvement in the antibacterial resistivity of medical screws. Here, two points should be mentioned: first, the antibacterial resistivity of the medical screw coated with only polymer (IS-P) was lower than that of the medical screw coated with only propolis (IS-P-7.5). This result shows that the gel coating elicited no significant improvements in antibacterial resistivity expected from choosing a biocompatible polymer (carbopol) as a carrier. Second, propolis needs a carrier polymer for it to be perfectly fixed on the medical screws; otherwise, it is easily released/lost during cleaning and/or sanitation steps. The carrier polymer also plays an important role in controlling the release profile of the antibacterial agents and propolis from the surface.

Furthermore, the amount of propolis loaded onto the coating layers also designated the antibacterial effects of the surface-coated medical screws. When the amount of propolis increased from 2.5% to 7.5%, the zone diameter also increased from 21.8 to 24.3 mm. The percentage improvements in the antibacterial feature of IS-7.5, IS-5.0, and IS-2.5 were 20.0%, 13.9%, and 7.8%, respectively, according to the screws coated with only propolis (IS-P-7.5), whereas these were 26.0%, 19.5%, and 13.0%, respectively, according to the screws coated with only gel (IS-P). If the same comparison was performed according to the blank disk, the improvements were 43.5%, 36.9%, and 28.7%, respectively. According to the result for the standard disk (penicillin-loaded disk with a zone diameter of 30.0 mm), the antibacterial effects of propolis-loaded medical screws were lower.

Some of the limitations of the study include that it is supported by animal experimental studies, and the antibacterial efficacy was evaluated with only one bacterial species.

## Conclusions

In our view, the gel and propolis combination significantly improved the antibacterial resistance of the medical screws, and the method developed in this study can be an alternative approach to increase the antibacterial resistance of medical implants owing to its simplicity, effectiveness, and biocompatibility.
